# Characteristics and comprehensiveness of adult HIV care and treatment programmes in Asia-Pacific, sub-Saharan Africa and the Americas: results of a site assessment conducted by the International epidemiologic Databases to Evaluate AIDS (IeDEA) Collaboration

**DOI:** 10.7448/IAS.17.1.19045

**Published:** 2014-12-15

**Authors:** Stephany N Duda, Amanda M Farr, Mary Lou Lindegren, Meridith Blevins, C William Wester, Kara Wools-Kaloustian, Didier K Ekouevi, Matthias Egger, Jennifer Hemingway-Foday, David A Cooper, Richard D Moore, Catherine C McGowan, Denis Nash, the International Epidemiologic Databases to Evaluate AIDS (IeDEA) Collaboration

**Affiliations:** 1 Department of Biomedical Informatics, Vanderbilt School of Medicine, Nashville, TN, USA; 2 Department of Life Sciences, Truven Health Analytics, Cambridge, MA, USA; 3 School of Public Health, City University of New York, New York, NY, USA; 4 Department of Pediatrics, Vanderbilt School of Medicine, Nashville, TN, USA; 5 Department of Health Policy, Vanderbilt School of Medicine, Nashville, TN, USA; 6 Department of Biostatistics, Vanderbilt School of Medicine, Nashville, TN, USA; 7 Vanderbilt Institute for Global Health (VIGH), Nashville, TN, USA; 8 Department of Medicine, Division of Infectious Diseases, Vanderbilt School of Medicine, Nashville, TN, USA; 9 Department of Medicine, Indiana University School of Medicine, Indianapolis, IN, USA; 10 Université de Bordeaux, ISPED, centre Inserm U897 Épidémiologie-Biostatistique, 33000 Bordeaux, France; 11 Programme PAC-CI, Site ANRS de Côte d’Ivoire, Abidjan, Côte d’Ivoire; 12 Université de Lomé, Faculté des Sciences de la Santé, Départment de Santé Publique, Lomé, Togo; 13 Institute of Social and Preventive Medicine (ISPM), University of Bern, Bern, Switzerland; 14 Research Triangle Institute, Raleigh, NC, USA; 15 Kirby Institute, University of New South Wales, Sydney, Australia; 16 Department of Medicine, Johns Hopkins University School of Medicine, Baltimore, MD, USA; 17 Department of Epidemiology, Johns Hopkins Bloomberg School of Public Health Baltimore, MD, USA; 18 Epidemiology and Biostatistics Program, Hunter College, City University of New York, New York, NY, USA; 19 Doctor of Public Health Programs (CUNY SPH), School of Public Health, City University of New York, New York, NY, USA

**Keywords:** HIV/AIDS, HIV care capacity, clinic characteristics, comprehensive care, resource-limited settings

## Abstract

**Introduction:**

HIV care and treatment programmes worldwide are transforming as they push to deliver universal access to essential prevention, care and treatment services to persons living with HIV and their communities. The characteristics and capacity of these HIV programmes affect patient outcomes and quality of care. Despite the importance of ensuring optimal outcomes, few studies have addressed the capacity of HIV programmes to deliver comprehensive care. We sought to describe such capacity in HIV programmes in seven regions worldwide.

**Methods:**

Staff from 128 sites in 41 countries participating in the International epidemiologic Databases to Evaluate AIDS completed a site survey from 2009 to 2010, including sites in the Asia-Pacific region (*n=*20), Latin America and the Caribbean (*n=*7), North America (*n=*7), Central Africa (*n=*12), East Africa (*n=*51), Southern Africa (*n=*16) and West Africa (*n=*15). We computed a measure of the comprehensiveness of care based on seven World Health Organization-recommended essential HIV services.

**Results:**

Most sites reported serving urban (61%; region range (rr): 33–100%) and both adult and paediatric populations (77%; rr: 29–96%). Only 45% of HIV clinics that reported treating children had paediatricians on staff. As for the seven essential services, survey respondents reported that CD4+ cell count testing was available to all but one site, while tuberculosis (TB) screening and community outreach services were available in 80 and 72%, respectively. The remaining four essential services – nutritional support (82%), combination antiretroviral therapy adherence support (88%), prevention of mother-to-child transmission (PMTCT) (94%) and other prevention and clinical management services (97%) – were uniformly available. Approximately half (46%) of sites reported offering all seven services. Newer sites and sites in settings with low rankings on the UN Human Development Index (HDI), especially those in the President's Emergency Plan for AIDS Relief focus countries, tended to offer a more comprehensive array of essential services. HIV care programme characteristics and comprehensiveness varied according to the number of years the site had been in operation and the HDI of the site setting, with more recently established clinics in low-HDI settings reporting a more comprehensive array of available services. Survey respondents frequently identified contact tracing of patients, patient outreach, nutritional counselling, onsite viral load testing, universal TB screening and the provision of isoniazid preventive therapy as unavailable services.

**Conclusions:**

This study serves as a baseline for on-going monitoring of the evolution of care delivery over time and lays the groundwork for evaluating HIV treatment outcomes in relation to site capacity for comprehensive care.

## Introduction

By the end of 2012, an estimated 35 million people worldwide were living with HIV, of whom 71% resided in sub-Saharan Africa [1]. International donors and individual countries are striving to scale up combination antiretroviral therapy (ART) to reach the 28.3 million people eligible for treatment in 2013 [1]. HIV care and treatment programmes, particularly in resource-limited settings where the vast majority of eligible adults reside, are expanding to support the implementation of HIV prevention, care and treatment interventions. However, programme coverage remains insufficient and is not evenly distributed [2]. Monitoring key programme outcomes (e.g. immunologic, virologic and clinical response to treatment; survival; adherence to treatment; and retention in care) and programme targets (e.g. number of patients enrolled in care and initiating ART) has provided insights into the successes and challenges of HIV treatment scale-up [3–12], just as intensive reviewing of data from national HIV response plans and monitoring and evaluation systems has revealed the challenges of programme implementation in resource-limited settings [13,14].

Observational studies relate site and programme characteristics to improved patient and programme outcomes [13,15], including higher CD4+ cell count at the time of ART initiation [16,17] and improved ART medication adherence [18], service utilization [19] and loss to follow-up in HIV care [20]. Such analyses are an important part of the larger agenda of rigorous evaluation and implementation science in any large-scale service or prevention programme [13,21,22]. Understanding programme characteristics, their evolution over time and how these characteristics may influence patient outcomes such as retention in care, immunodeficiency at ART initiation and response to ART strengthens the process of constructing and implementing more effective HIV care and treatment programmes.

The comprehensiveness of HIV care services may also play an important role in influencing patient and programme outcomes, but it has not been well described. Comprehensive HIV care depends on the provision of diverse services, including HIV prevention services, HIV counselling and testing, prevention of illness, management of opportunistic infections and comorbidities, ART adherence support, patient monitoring on ART and palliative care [23–25]. The World Health Organization (WHO) has identified these recommended services as HIV priority interventions [2].

In this analysis, we outlined the facility and programme characteristics in a large global network of HIV clinical sites and assessed the “comprehensiveness” of HIV prevention, care and treatment service availability. We compared the comprehensive care capacity of established, resource-rich sites to that of newer sites in resource-limiting settings.

## Methods

The International epidemiologic Databases to Evaluate AIDS (IeDEA) consortium (http://www.iedea.org) is a research network of HIV care and treatment programmes in seven geographical regions: Asia-Pacific, the Caribbean/Latin America, North America, Central Africa, East Africa, Southern Africa and West Africa [26–30]. Each IeDEA region has an independent data centre and governance structure, although the regions collaborate on cross-region projects. IeDEA is funded by the US National Institutes of Health (NIH) to address key clinical and operational research questions that require data on large numbers of patients receiving care across a spectrum of clinical care settings.

### Survey development

IeDEA investigators developed a 164-item site survey to elicit information on site characteristics including the following:

- the facility housing the HIV clinic (facility level, teaching affiliation and public or private sector);
- the HIV care clinic (number of clinic days per week that the clinic provides HIV services, year ART services began and current waiting list for ART);
- the patient population (adult/paediatric and urban/rural patients);
- components of the HIV care programme (voluntary counselling and testing, HIV prevention services, prevention of mother-to-child transmission (PMTCT) of HIV and nutritional services);
- available support services (availability of ART adherence services, outreach to patients who miss appointments and a peer educator programme);
- available laboratory services (CD4+ cell count: onsite, offsite or not available; CD4+ cell count turnaround time; HIV-1 viral load: onsite, offsite or not available; and labs to monitor adverse events);
- prevention, diagnosis and management of co-infections (tuberculosis (TB) and malaria) and malignancies;
- supply chain reliability (frequency of ART medication and CD4+ cell count reagent stock-outs);
- the ART pharmacy; and
- the cadres of HIV clinic staff.

English and French versions of the IeDEA site survey were available either online or as paper-based instruments. The online version was implemented using REDCap, a secure, web-based application designed and hosted at Vanderbilt University to support data capture for research studies [31]. The paper-based survey was translated from English into French, Spanish and Portuguese by professional translators at the NIH Clinical Center in Bethesda, United States.

### Data collection

All seven IeDEA regions agreed to participate in the study. Data managers from each IeDEA region distributed a link to the web-based site assessment survey and a PDF of the paper survey to clinical staff at the adult-only or combined adult-paediatric care clinics within their geographic region. Any surveys returned on paper were entered into REDCap and verified by the regional data teams. The Southern Africa region completed a subset of the survey questions, soliciting data on facility characteristics and opportunistic infection management, but not on other programme characteristics or laboratory capacity. Southern Africa used the WHO DataCol software package (https://extranet.who.int/datacol/home.asp), which was an established data collection tool in their region, and entered data in duplicate into REDCap.

The site assessment was conducted in all seven IeDEA regions from August 2009 to February 2010. The sites and coordinating centres for all IeDEA regions had Institutional Review Board approvals in place that permitted the collection of such operational data through this site assessment survey. The protocol was reviewed by Columbia University Institutional Review Board and received nonhuman subject research determination, as the subjects of data collection were facilities and not individuals.

### Comprehensiveness assessment

Authors, including HIV clinicians, identified questions on the already-finalized survey that addressed essential HIV comprehensive care services as described by WHO and published literature [2,23,24]. We assembled these questions into a comprehensiveness metric based on the site-level availability of seven essential services: (1) ART adherence support, defined as providing on a routine basis one-on-one adherence counselling, reminders and review of medication pickup; (2) CD4+ cell count testing onsite or offsite; (3) HIV prevention services: specifically the availability of HIV testing and counselling, the provision of co-trimoxazole (TMP-SMX) for *Pneumocystis jiroveci* (formerly *carinii*) pneumonia (PCP) prophylaxis and at least two other prevention services (education on high-risk behaviours, screening for substance abuse and sexually transmitted infections, family planning, post-exposure prophylaxis and/or adult male circumcision); (4) PMTCT services, either onsite or offsite and linked with onsite care; (5) nutritional counselling and support, including provision of multivitamins, mineral supplements and nutritional “treatment” for malnutrition, or an onsite nutritionist; (6) universal screening for TB symptoms; and (7) community outreach and contact tracing for ART-treated adults with missed clinic appointments. These services were included in the Priority Interventions recommended by WHO [2]. We evaluated the availability of these seven essential HIV comprehensive care services for every clinic that completed all the associated survey questions.

Sites were grouped into comprehensiveness categories of *low* (3–5 essential services), *medium* (6 essential services) or *high* (all 7 essential services). We evaluated the availability of essential services according to site characteristics, such as urban or rural patient population, year of first ART availability for adults, country rank on the 2010 UN Human Development Index (HDI) (rankings that take into account per capita income as well as life expectancy) [32] and US President's Emergency Plan for AIDS Relief (PEPFAR) focus country status as of 2008 [33].

### Statistical considerations

Data from each of the seven regions were merged, cleaned and analysed using Microsoft Excel and the R statistical software package. Analyses included descriptive statistics and frequency calculations. Analysis scripts are available on the Vanderbilt University Department of Biostatistics wiki (http://biostat.mc.vanderbilt.edu/ArchivedAnalyses). Descriptive statistics of programme and facility characteristics are presented by region and by comprehensiveness category.

## Results

**Figure 1:**
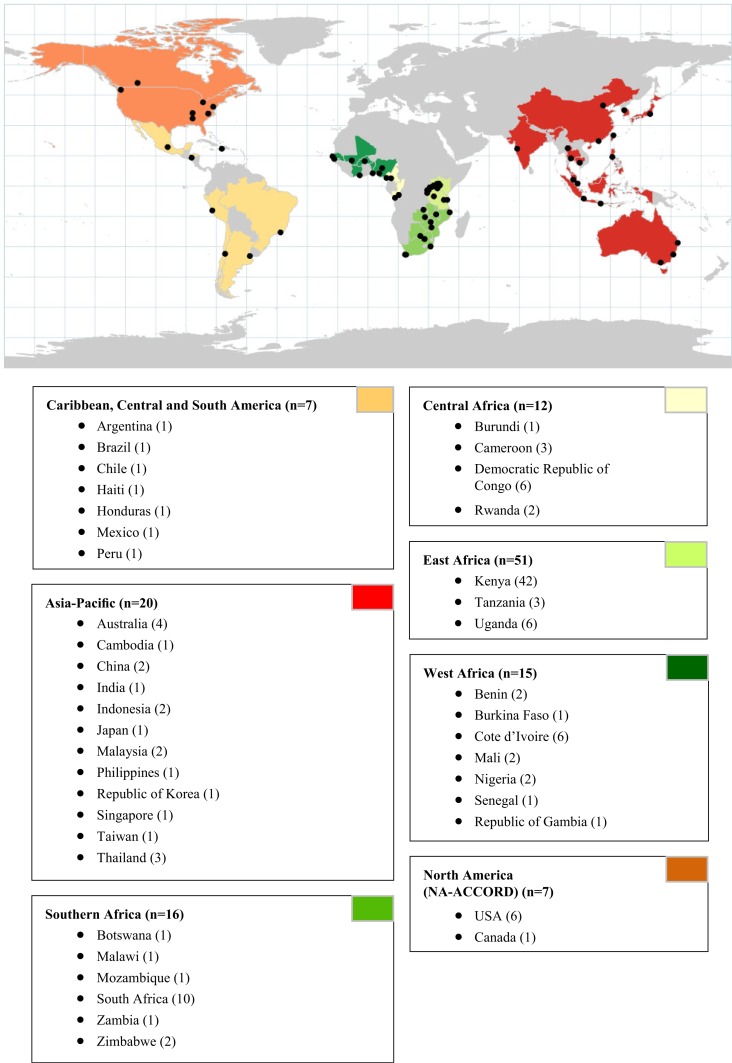
Geographical distribution of the HIV treatment programmes from the IeDEA network that participated in the site assessment.

One hundred and thirty-three HIV care and treatment sites within IeDEA were approached for participation in this study, and 128 of 133 (96%) completed the site survey. The number of participating sites per region varied from seven each in Caribbean/Latin America and North America to 51 in East Africa. Figure 1 depicts the geographic distribution of the sites, and site characteristics are outlined in Table 1.

**Table 1:** Adult HIV care and treatment facility characteristics by region

	North America *N=*7	Latin America *N=*7	Asia-Pacific *N=*20	Central Africa *N=*12	East Africa *N=*51	Southern Africa *N=*16	West Africa *N=*15	All Regions *N=*128
Patient population, *n* (%)								
Urban	6 (86%)	7 (100%)	17 (85%)	8 (67%)	17 (33%)	14 (88%)	9 (60%)	78 (61%)
Rural	–	–	–	–	14 (27%)	2 (13%)	–	16 (13%)
Mixed	1 (14%)	–	3 (15%)	4 (33%)	20 (39%)	–	6 (40%)	34 (27%)
Patients seen in clinic, *n* (%)								
Adults only	5 (71%)	3 (43%)	13 (65%)	1 (8%)	2 (4%)	1 (6%)	5 (33%)	30 (23%)
Adults and children	2 (29%)	4 (57%)	7 (35%)	11 (92%)	49 (96%)	15 (94%)	10 (67%)	98 (77%)
Level of facility, *n* (%)^a^,^b^								
Missing^b^	–	–	–	–	2 (4%)	–	–	2 (2%)
Primary	2 (29%)	1 (14%)	1 (5%)	7 (58%)	24 (49%)	9 (56%)	4 (27%)	48 (38%)
Secondary	–	–	1 (5%)	1 (8%)	18 (37%)	4 (25%)	2 (13%)	26 (21%)
Tertiary	5 (71%)	6 (86%)	18 (90%)	4 (33%)	7 (14%)	3 (19%)	9 (60%)	52 (41%)
Type of facility, *n* (%)^b^								
Missing	–	–	–	1 (8%)	2 (4%)	–	–	3 (2%)
Public	4 (57%)	6 (86%)	18 (90%)	5 (45%)	44 (90%)	10 (63%)	12 (80%)	99 (79%)
Private	3 (43%)	1 (14%)	2 (10%)	6 (55%)	5 (10%)	6 (38%)	3 (20%)	26 (21%)
Year ART services began, *n* (%)								
<2000	7 (100%)	3 (43%)	17 (85%)	–	1 (2%)	2 (13%)	4 (27%)	34 (27%)
2000–2004	–	3 (43%)	2 (10%)	4 (33%)	17 (33%)	11 (69%)	10 (67%)	47 (37%)
2005 and later	–	1 (14%)	1 (5%)	7 (58%)	28 (55%)	2 (13%)	–	39 (30%)
Number of sites with provider category on staff, *n* (%)								
Physicians	7 (100%)	7 (100%)	20 (100%)	12 (100%)	31 (61%)	15 (94%)	14 (93%)	106 (83%)
Paediatricians	2 (29%)	4 (57%)	9 (45%)	2 (17%)	14 (27%)	9 (56%)	7 (47%)	47 (37%)
Mid-level providers^c^	6 (86%)	2 (29%)	8 (40%)	4 (33%)	44 (86%)	5 (31%)	3 (20%)	72 (56%)
Nurses/midwives	7 (100%)	7 (100%)	19 (95%)	8 (67%)	45 (88%)	16 (100%)	12 (80%)	114 (89%)
Nursing assistants^d^	6 (86%)	4 (57%)	11 (55%)	6 (50%)	10 (20%)		5 (33%)	42 (33%)
Lay health workers, adherence counsellors or outreach workers	7 (100%)	6 (86%)	14 (70%)	10 (83%)	44 (86%)	14 (88%)	13 (87%)	108 (84%)
Pharmacists	7 (100%)	5 (71%)	18 (90%)	5 (42%)	12 (24%)	13 (81%)	12 (80%)	72 (56%)
Pharmacy assistants	5 (71%)	5 (71%)	8 (40%)	7 (58%)	38 (75%)	13 (81%)	12 (80%)	88 (69%)
Nutritionists^d^	6 (86%)	3 (43%)	10 (50%)	5 (42%)	28 (55%)		5 (33%)	57 (45%)
Data capturers	7 (100%)	6 (86%)	17 (85%)	11 (92%)	24 (47%)	13 (81%)	14 (93%)	92 (72%)

a Primary facilities are health centres or clinics. Secondary facilities are district or provincial hospitals. Tertiary facilities are teaching or national reference hospitals. Some sites reported more than one facility type. When possible, their responses were disambiguated based on their reported mean number of HIV patients seen daily;

b Ppercentages are computed using the number of facilities with a non-missing value;

c mid-level providers include clinical officers, nurse practitioners and physician assistants;

d Southern Africa did not query its sites about these provider categories.

Most of the 128 clinics reported serving urban populations (61%). This pattern was consistent across regions with the exception of East Africa (33%). All clinics provided HIV care to adult patients and the majority of clinics (77%) also provided care to children; across regions, this ranged from 29% in North America to 96% in East Africa. Most HIV clinics defined themselves as being located within primary care facilities (38%; region range (rr): 5–58%) or tertiary care facilities (41%; rr: 14–90%). Only East Africa had a substantial proportion of HIV clinics in secondary-level health facilities (37%). Most facilities were publicly funded (79%), except in Central Africa (44%). Most clinics in North America, Caribbean/Latin America and Asia-Pacific began offering HIV treatment before 2000, whereas the clinics in the less resourced regions of sub-Saharan Africa began offering ART more recently (Table 1).

Staff capacity varied by IeDEA region: 83% of HIV clinics reported having onsite physicians, ranging from 61% in East Africa to 100% in North America, Caribbean/Latin America, Asia-Pacific and Central Africa. However, paediatricians were only available at 37% of all HIV clinics (from 17% in Central Africa to 57% in Caribbean/Latin America) and at 45% of HIV clinics that reported treating children. Sites reported that mid-level providers (clinical officers, nurse practitioners and physician assistants) were available at 56% of HIV clinics across all regions, ranging from 20% in West Africa to 86% in North America and East Africa.

### Programme characteristics and clinical services

Table 2 lists characteristics of the HIV care and treatment programmes in six IeDEA regions, as self-reported by participating sites. Two HIV prevention services were almost universally available: HIV counselling and testing (97%; rr: 86–100%) and HIV disclosure counselling (94%; rr: 83–100%). Condoms were less commonly provided by HIV clinics in West Africa (47%) than in other regions (range: 70–100%), while only North America routinely provided drug and alcohol abuse screening (100%, vs. 14–60%) and substance use and harm reduction education (100%, vs. 33–65%). Adult male circumcision was offered at 38% of all clinics. East Africa was an exception with 65% of sites offering adult male circumcision. co-trimoxazole (or the equivalent, including Septra™ or Bactrim) prophylaxis was available at 97% of sites (rr: 92–100%). One hundred and seven sites (95%) reported available patient support services, especially patient support groups and peer educator programmes, available at 88% (rr: 86–100%) and 67% (rr: 50–75%) of clinics, respectively. The vast majority (94%) of sites (rr: 57–100%) reported conducting at least one form of outreach and tracking for ART-treated adults with missed clinic visits; and 98% (rr: 93–100%) provided one or more ART adherence support services on a routine basis. According to survey responses, nutritional counselling (70%; rr: 63–86%) and micronutrient supplementation (54%; rr: 40–60%) were not as universally available although relatively uniform across regions. PMTCT services were reported to be available onsite at 77% and offsite at 11% of all adult HIV clinics.

**Table 2:** Adult HIV programme characteristics and laboratory capacity reported by region

	Total *n* (%)						
	
	North America *N=*7	Latin America *N=*7	Asia-Pacific *N=*20	Central Africa *N=*12	East Africa *N=*51	West Africa *N=*15	Total *N=*112^a^
Availability of prevention services							
HIV counselling and testing	6 (86%)	6 (86%)	20 (100%)	12 (100%)	51 (100%)	14 (93%)	109 (97%)
Counselling regarding disclosure to partners	7 (100%)	6 (86%)	18 (90%)	10 (83%)	51 (100%)	13 (87%)	105 (94%)
Education on sexual behaviour changes/safer sex	7 (100%)	5 (71%)	19 (95%)	11 (92%)	48 (94%)	8 (53%)	98 (88%)
Provision of condoms	7 (100%)	6 (86%)	14 (70%)	9 (75%)	47 (92%)	7 (47%)	90 (80%)
Family planning for PMTCT	6 (86%)	5 (71%)	15 (75%)	5 (42%)	47 (92%)	7 (47%)	85 (76%)
Referral for onsite screening for sexually transmitted infections	7 (100%)	5 (71%)	17 (85%)	6 (50%)	45 (88%)	8 (53%)	88 (79%)
Education on high-risk substance use behaviours and harm reduction practices	7 (100%)	3 (43%)	13 (65%)	5 (42%)	32 (63%)	5 (33%)	65 (58%)
Screening for drug and alcohol abuse and referral to treatment	7 (100%)	1 (14%)	12 (60%)	3 (25%)	17 (33%)	3 (20%)	43 (38%)
Post-exposure prophylaxis (PEP)	6 (86%)	4 (57%)	19 (95%)	10 (83%)	42 (82%)	12 (80%)	93 (83%)
Male circumcision for adults	2 (29%)	–	3 (15%)	2 (17%)	33 (65%)	3 (20%)	43 (38%)
Co-trimoxazole prophylaxis	7 (100%)	7 (100%)	20 (100%)	11 (92%)	50 (98%)	14 (93%)	109 (97%)
Other	–	1 (14%)	–	1 (8%)	8 (16%)	1 (7%)	11 (10%)
None of the above	–	–	–	–	2 (4%)	1 (7%)	3 (3%)
Availability of support services							
Support groups	6 (86%)	7 (100%)	16 (80%)	11 (92%)	46 (90%)	13 (87%)	99 (88%)
Peer educator programme	5 (71%)	5 (71%)	13 (65%)	6 (50%)	38 (75%)	8 (53%)	75 (67%)
Outreach programme (pre-ART and/or ART)	5 (71%)	3 (43%)	10 (50%)	10 (83%)	44 (86%)	13 (87%)	85 (76%)
Other	1 (14%)	1 (14%)	–	1 (8%)	2 (4%)	1 (7%)	6 (5%)
None of the above	–	–	2 (10%)	–	1 (2%)	2 (13%)	5 (5%)
Outreach methods							
Phone call	6 (86%)	2 (29%)	18 (90%)	9 (75%)	36 (71%)	12 (80%)	83 (74%)
Letter sent	5 (71%)	–	6 (30%)	–	–	–	11 (10%)
Home visit	–	2 (29%)	6 (30%)	11 (92%)	48 (94%)	12 (80%)	79 (71%)
Consult pharmacy	–	3 (43%)	6 (30%)	3 (25%)	15 (29%)	3 (20%)	30 (27%)
Check hospital records	1 (14%)	–	6 (30%)	4 (33%)	24 (47%)	2 (13%)	37 (33%)
None of the above	–	3 (43%)	1 (5%)	1 (8%)	1 (2%)	1 (7%)	7 (6%)
Availability of ART adherence support services							
Counselling (one-on-one)	7 (100%)	7 (100%)	19 (95%)	11 (92%)	50 (98%)	14 (93%)	108 (96%)
Counselling (group)	3 (43%)	3 (43%)	4 (20%)	9 (75%)	45 (88%)	11 (73%)	75 (67%)
Educational materials^b^	6 (86%)	3 (43%)	15 (75%)	7 (58%)	29 (57%)	4 (27%)	64 (57%)
Reminder tools^c^	7 (100%)	4 (57%)	15 (75%)	10 (83%)	45 (88%)	7 (47%)	88 (79%)
Routine review of medication pick-up	5 (71%)	5 (71%)	9 (45%)	5 (42%)	38 (75%)	9 (60%)	71 (63%)
None of the above	–	–	–	–	1 (2%)	1 (7%)	2 (2%)
Availability of nutritional services for adult patients							
Nutritional counselling	6 (86%)	6 (86%)	15 (75%)	9 (75%)	32 (63%)	10 (67%)	78 (70%)
Any nutrition support	6 (86%)	7 (100%)	15 (75%)	11 (92%)	51 (100%)	12 (80%)	102 (91%)
Micronutrient supplementation	4 (57%)	4 (57%)	8 (40%)	6 (50%)	29 (57%)	9 (60%)	60 (54%)
Nutritional “treatment” for severely malnourished adults	4 (57%)	1 (14%)	9 (45%)	4 (33%)	37 (73%)	4 (27%)	59 (53%)
Food rations	–	2 (29%)	–	5 (42%)	28 (55%)	8 (53%)	43 (38%)
None	–	–	4 (20%)	–	–	2 (13%)	6 (5%)
Missing	1 (14%)	–	–	–	–	1 (7%)	2 (2%)
Availability of PMTCT services							
Onsite	5 (71%)	6 (86%)	13 (65%)	7 (58%)	46 (90%)	9 (60%)	86 (77%)
Offsite	–	–	3 (15%)	3 (25%)	4 (8%)	2 (13%)	12 (11%)
None	–	1 (14%)	4 (20%)	2 (17%)	–	2 (13%)	9 (8%)
Missing	2 (29%)	–	–	–	1 (2%)	2 (13%)	5 (4%)
Laboratory capacity							
CD4^+^ cell count testing							
Onsite	5 (71%)	6 (86%)	17 (85%)	9 (75%)	19 (37%)	10 (67%)	66 (59%)
Offsite	2 (29%)	1 (14%)	3 (15%)	3 (25%)	30 (59%)	3 (20%)	42 (38%)
Not available	–	–	–	–	1 (2%)	–	1 (1%)
Missing	–	–	–	–	1 (2%)	2 (13%)	3 (3%)
CD4 turnaround time^d^							
Turnaround days (median, IQR)	1 (1–2.5)	7 (2–10)	2 (1–5.5)	2 (1.75–4.25)	7 (1–14)	8 (3.5–11)	3 (1–10)
Missing (*n*,%)	–	2 (29%)	1 (5%)	1 (8%)	9 (18%)	4 (27%)	17 (15%)
CD4 reagent stock-outs in last 12 months^d^							
Yes	–	1 (14%)	1 (5%)	8 (67%)	14 (27%)	2 (13%)	26 (23%)
No	6 (86%)	5 (71%)	18 (90%)	1 (8%)	29 (57%)	10 (67%)	69 (62%)
Missing	1 (14%)	1 (14%)	1 (5%)	3 (25%)	7 (14%)	3 (20%)	16 (14%)
HIV RNA PCR testing							
Onsite	5 (71%)	4 (57%)	14 (70%)	–	6 (12%)	5 (33%)	34 (30%)
Offsite	2 (29%)	2 (29%)	3 (15%)	7 (58%)	37 (73%)	7 (47%)	58 (52%)
Not available	–	–	–	5 (42%)	5 (10%)	1 (7%)	11 (10%)
Missing	–	1 (14%)	3 (15%)	–	3 (6%)	2 (13%)	9 (8%)

a The 16 sites from Southern Africa are excluded as their survey did not contain these questions;

b educational materials include written and pictorial patient education material and educational videotapes;

c reminder tools include appointment slips, calendars, checklists or other reminders, alarm clocks, wrist watches and beepers;

d sites reporting no CD4+ measurement capabilities are excluded from the N in these calculations.

### HIV laboratory capacity

All but four clinics reported having CD4+ cell count testing available either onsite (59%; rr: 37–86%) or offsite (38%; rr: 14–59%), with a median turnaround time of three days (interquartile range: 1–10) (Table 2). Additionally, 23% (rr: 0–67%) reported experiencing a CD4+ cell count reagent stock-out in the 12 months preceding the survey. Overall, 82% of sites (rr: 58–100%) reported having access to HIV-1 plasma RNA (viral load) testing, although onsite viral load testing was uncommon (30%, rr: 0–71%).

### ART provision

Across all seven IeDEA regions, 34% (rr: 0–50%) of clinics reported having a waiting list for ART patients, as shown in Table 3. Only 82 of the 128 clinics (64%; rr: 33–100%) reported no ARV medication stock-outs in the 12 months preceding the survey.

**Table 3:** Antiretroviral provision and management of tuberculosis and malaria

	Total *n* (%)							
	
	North America *N=*7	Latin America *N=*7	Asia-Pacific *N=*20	Central Africa *N=*12	East Africa *N=*51	Southern Africa *N=*16	West Africa *N=*15	All regions *N=*128
ART provision								
Current waiting list for ART patients (*N*,%)								
Yes	–	–	2 (10%)	6 (50%)	23 (45%)	5 (31%)	7 (47%)	43 (34%)
No	–	7 (100%)	18 (90%)	6 (50%)	28 (55%)	11 (69%)	7 (47%)	77 (60%)
Missing	7 (100%)	–	–	–	–	–	1 (7%)	8 (6%)
Duration of ART medication stock-outs in last 12 months								
1 day only	–	–	1 (5%)	1 (8%)	1 (2%)	–	–	3 (2%)
2–7 days	–	–	1 (5%)	2 (17%)	1 (2%)	–	2 (13%)	6 (5%)
8–21 days	–	1 (14%)	–	–	4 (8%)	–	2 (13%)	7 (5%)
> 21 days	–	1 (14%)	–	3 (25%)	2 (4%)	–	2 (13%)	8 (6%)
Duration unknown	–	1 (14%)	–	1 (8%)	1 (2%)	–	3 (20%)	12 (9%)
No stock-outs	–	4 (57%)	17 (85%)	5 (42%)	41 (80%)	6 (38%)	5 (33%)	82 (64%)
Missing	7 (100%)	–	1 (5%)	–	1 (2%)	10 (63%)	1 (7%)	10 (8%)
Tuberculosis								
Location of TB treatment services								
Within onsite HIV care and treatment facility	1 (14%)	2 (29%)	11 (55%)	4 (33%)	18 (35%)	14 (88%)	11 (73%)	61 (48%)
Onsite TB clinic	1 (14%)	5 (71%)	6 (30%)	2 (17%)	24 (47%)	–	1 (7%)	39 (30%)
Offsite TB clinic	5 (71%)	–	3 (15%)	5 (42%)	5 (10%)	2 (13%)	2 (13%)	22 (17%)
Missing	–	–	–	1 (8%)	4 (8%)	–	1 (7%)	6 (5%)
TB screening^a^								
Ask about symptoms as standard part of patient history	4 (57%)	6 (86%)	11 (55%)	10 (83%)	39 (76%)	16 (100%)	12 (80%)	98 (77%)
Formal questionnaire	1 (14%)	1 (14%)	2 (10%)	5 (42%)	30 (59%)	7 (44%)	6 (40%)	52 (41%)
Tuberculin skin test	7 (100%)	6 (86%)	6 (30%)	3 (25%)	2 (4%)	7 (44%)	3 (20%)	34 (27%)
DOTS for adult TB patients								
First 2 months	–	1 (14%)	1 (5%)	4 (33%)	19 (37%)	6 (38%)	6 (40%)	37 (29%)
Entire period	4 (57%)	4 (57%)	9 (45%)	1 (8%)	13 (25%)	6 (38%)	6 (40%)	43 (34%)
No	3 (43%)	2 (29%)	10 (50%)	3 (25%)	12 (24%)	4 (25%)	2 (13%)	36 (28%)
Missing	–	–	–	4 (33%)	7 (14%)	–	1 (7%)	12 (9%)
Availability of isoniazid prophylaxis								
For all patients	–	3 (43%)	4 (20%)	1 (8%)	20 (39%)	2 (13%)	2 (13%)	32 (25%)
For some patients	–	4 (57%)	11 (55%)	3 (25%)	5 (10%)	13 (81%)	1 (7%)	37 (29%)
Not available	–	–	5 (25%)	7 (58%)	21 (41%)	1 (6%)	12 (80%)	46 (36%)
Missing	7 (100%)	–	–	1 (8%)	5 (10%)	–	–	13 (10%)
Malaria								
Malaria diagnostic methods								
Presumptive diagnosis	–	1 (14%)	2 (10%)	5 (42%)	35 (69%)	3 (19%)	9 (60%)	55 (43%)
Thick smear	–	5 (71%)	16 (80%)	12 (100%)	45 (88%)	11 (69%)	10 (67%)	99 (77%)
Rapid test	–	1 (14%)	6 (30%)	1 (8%)	13 (25%)	9 (56%)	6 (40%)	36 (28%)
Other	–	–	2 (10%)	–	–	–	1 (7%)	3 (2%)
Not applicable	–	2 (29%)	2 (10%)	–	–	3 (19%)	–	7 (5%)
Missing	7 (100%)	–	–	–	–	1 (6%)	1 (7%)	9 (7%)
Distribution of free bed nets^b^								
All patients	–	–	1 (5%)	–	7 (14%)	–	2 (13%)	10 (8%)
Targeted distribution	–	–	1 (5%)	2 (17%)	32 (63%)	–	1 (7%)	36 (28%)
Not distributed/not applicable	–	7 (100%)	18 (90%)	10 (83%)	11 (22%)	–	11 (73%)	57 (45%)
Missing	7 (100%)	–	–	–	1 (2%)	16 (100%)	1 (7%)	25 (20%)

a TB screening includes screening done on all patients only;

b one site reported that it did not distribute free bed nets, while also saying it distributed free bed nets to pregnant women and paediatric patients under age 5. This site was only included under targeted distribution.

### Screening, diagnosis and management of TB and malaria

TB symptom screening was conducted among the HIV clinic populations at 77% of sites, with variability across regions ranging from 55% in Asia-Pacific to 100% in Southern Africa. However, only 41% of sites (rr: 10–59%) reported using a formal TB screening questionnaire. TB skin testing was available at only 27% of sites (ranging from 4% in East Africa to 100% in North America). Isoniazid preventative therapy (IPT) was available for all patients living with HIV at only 25% (rr: 0–43%) of clinics and was not available at all in 36% (rr: 0–80%) of clinics.

TB treatment was located within the HIV care and treatment facility in 48% of sites (ranging from 14% in North America to 88% in Southern Africa) and in an onsite TB clinic in an additional 30% (rr: 0–71%) (Table 3). The majority of participating clinics (63%; rr: 41–80%) instituted directly observed TB therapy (TB-DOTS) for TB treatment in adult patients. Absence of TB-DOTS was most common in clinics responding from North America (43%) and Asia-Pacific (50%).

The provision of bed nets by sites to patients for malaria prevention was uncommon in all regions (36%) except in East Africa (77% of sites). Sites that reported managing malaria diagnosed most malaria cases using thick smears (77%; rr: 67–100%).

### Comprehensiveness of HIV care

Table 4 summarizes the distribution of seven essential HIV care services by IeDEA region. Comprehensiveness measures were calculated for the 93 sites (73%) for which complete survey data were available. Sites with missing data, which included all sites in Southern Africa, were excluded from the analysis, although sensitivity analyses were performed that included sites with partial data. Twenty of the 93 sites (22%) offered only 3–5 essential services (*low*); 30 sites (32%) offered 6 essential services (*medium*); and 43 sites (46%) were fully comprehensive, offering all 7 essential services (*high*). CD4+ cell count testing was available either onsite or offsite at all sites (100%), whereas universal TB screening and community outreach/tracking of adults on ART services were offered least often at 80 and 72%, respectively. Nutritional support (82%), ART adherence support (88%), PMTCT services (94%) and prevention and clinical management services (97%) were available more frequently.

**Table 4:** Reported distribution of HIV services by IeDEA region^a^

	Service by region			
	
	Offered	Not offered	Missing	% Offered (of non-missing)
North America (*n=*7)				
ART adherence	6	1	0	86
Nutritional support	7	0	0	100
PMTCT	5	1	1	83
CD4 testing	7	0	0	100
TB screening	4	3	0	57
Prevention	6	1	0	86
Outreach	5	2	0	71
Latin America (*n=*7)				
ART adherence	7	0	0	100
Nutritional support	4	3	0	57
PMTCT	6	1	0	86
CD4 testing	7	0	0	100
TB screening	6	1	0	86
Prevention	5	2	0	71
Outreach	3	4	0	43
Asia-Pacific (*n=*20)				
ART adherence	18	2	0	90
Nutritional support	15	5	0	75
PMTCT	17	3	0	85
CD4 testing	20	0	0	100
TB screening	11	9	0	55
Prevention	20	0	0	100
Outreach	9	11	0	45
Central Africa (*n=*12)				
ART adherence	9	3	0	75
Nutritional support	8	1	3	89
PMTCT	10	2	0	83
CD4 testing	12	0	0	100
TB screening	10	1	1	91
Prevention	12	0	0	100
Outreach	6	6	0	50
East Africa (*n=*51)				
ART adherence	41	8	2	84
Nutritional support	46	5	0	90
PMTCT	50	1	0	98
CD4 testing	49	1	1	98
TB screening	39	6	6	87
Prevention	51	0	0	100
Outreach	44	6	1	88
Vest Africa (*n=*17)				
ART adherence	11	2	2	85
Nutritional support	10	4	1	71
PMTCT	12	1	2	92
CD4 testing	15	0	2	100
TB screening	12	3	0	80
Prevention	15	0	1	100
Outreach	9	4	2	69
All regions (*n=*112)				
ART adherence	92	16	4	85
Nutritional support	90	18	4	83
PMTCT	100	9	3	92
CD4 testing	108	1	3	99
TB screening	82	23	7	78
Prevention	108	3	1	97
Outreach	76	33	3	70

a IeDEA Southern Africa is not represented in the essential services summary as data were not available for this region.

The characteristics of low, medium and highly comprehensive facilities are summarized in Table 5. Among the 43 fully comprehensive sites, 74% began ART provision in 2000–2009 with the vast majority (90%) providing services to both adults and children. Among 21 sites located in high-ranked or very high-ranked countries according to the 2010 HDI, 48% had low numbers of essential services where 43% performed patient outreach, 62% offered TB screening and 76% offered nutritional support programmes. Sites located in low-HDI countries receiving PEPFAR funding tended to offer more comprehensive services, with 94% of sites having 6–7 essential services.

**Table 5:** Facility characteristics by level of clinic comprehensiveness^a^

	Comprehensiveness (number of services)			
	
	Low (3–5) (% of row) *N=*20	Medium (6) (% of row) *N=*30	High (7) (% of row) *N=*43	All tiers^b^ (% of all sites) *N=*93
Region, *n* (%)				
Asia-Pacific	9 (45%)	7 (35%)	4 (20%)	20 (22%)
CCASAnet	3 (43%)	2 (29%)	2 (29%)	7 (8%)
Central Africa	1 (12%)	4 (50%)	3 (38%)	8 (9%)
East Africa	2 (5%)	11 (26%)	29 (69%)	42 (45%)
NA-ACCORD	2 (33%)	3 (50%)	1 (17%)	6 (6%)
West Africa	3 (30%)	3 (30%)	4 (40%)	10 (11%)
Patient population, *n* (%)				
Urban	16 (29%)	16 (29%)	23 (42%)	55 (59%)
Rural	–	3 (33%)	6 (67%)	9 (10%)
Mixed	4 (14%)	11 (38%)	14 (48%)	29 (31%)
Year of ART provision, *n* (%)				
Missing	1 (5%)	–	3 (7%)	4 (4%)
1985–1989	3 (27%)	5 (45%)	3 (27%)	11 (12%)
1990–1994	3 (60%)	2 (40%)	–	5 (6%)
1995–1999	5 (36%)	4 (29%)	5 (36%)	14 (16%)
2000–2004	6 (19%)	10 (31%)	16 (50%)	32 (36%)
2005–2009	2 (7%)	9 (33%)	16 (59%)	27 (30%)
Type of facility, *n* (%)				
Missing	1 (5%)	–	1 (2%)	2 (2%)
Private clinic	3 (25%)	5 (42%)	4 (33%)	12 (13%)
Public or government	16 (20%)	25 (32%)	38 (48%)	79 (87%)
Level of facility, *n* (%)				
Missing	1 (5%)	–	1 (2%)	2 (2%)
Primary	6 (16%)	11 (29%)	21 (55%)	38 (42%)
Secondary	–	3 (19%)	13 (81%)	16 (18%)
Tertiary	13 (35%)	16 (43%)	8 (22%)	37 (41%)
Patients seen in clinic, *n* (%)				
Adult only	14 (52%)	9 (33%)	4 (15%)	27 (29%)
Adults and children	6 (9%)	21 (32%)	39 (59%)	66 (71%)
PEPFAR country (2008), *n* (%)				
PEPFAR	3 (6%)	11 (22%)	36 (72%)	50 (54%)
No PEPFAR	17 (40%)	19 (44%)	7 (16%)	43 (46%)
HDI income category (2010), *n* (%)				
UN HDI-low	6 (10%)	18 (30%)	37 (61%)	61 (66%)
UN HDI-middle	4 (36%)	4 (36%)	3 (27%)	11 (12%)
UN HDI-high/very high	10 (48%)	8 (38%)	3 (14%)	21 (23%)
PEPFAR (2008) and HDI (2010), *n* (%)				
No PEPFAR: UN HDI-high/very high	10 (48%)	8 (38%)	3 (14%)	21 (23%)
No PEPFAR: UN HDI-low	3 (27%)	7 (64%)	1 (9%)	11 (12%)
No PEPFAR: UN HDI-middle	4 (36%)	4 (36%)	3 (27%)	11 (12%)
PEPFAR: UN HDI low	3 (6%)	11 (22%)	36 (72%)	50 (54%)

a IeDEA Southern Africa is not represented in the comprehensiveness analysis as data were not available for this region;

b Percentages are computed using the number of facilities with a non-missing value.

## Discussion

The survey of site capacity in this large global HIV care consortium revealed substantial regional variability in HIV care and treatment programmes and the comprehensiveness of HIV-associated services. Unexpectedly, IeDEA-participating HIV care and treatment sites in low-HDI settings were more likely to offer the full complement of essential services, compared to sites with medium or high HDI rankings. This difference appeared to be driven in part by low-HDI countries whose national programmes received PEPFAR support (72% of clinics reported having all seven essential services) compared with those low-HDI countries whose national programmes did not receive PEPFAR support (9% reported having all seven essential services). As international donor support for HIV care and treatment is waning, it will be important to track the continued availability of these comprehensive services over time, especially in the midst of and following full transitioning of care services to national authorities.

The lack of availability of all seven essential services at participating sites in middle- and high-HDI settings was surprising. The low prevalence of TB and malnutrition in such settings may lead to less emphasis on WHO-recommended services such as TB screening and nutritional support. This may also reflect the structure of how HIV care is provided in most high-HDI countries, where general patient care is offered through distributed, fee-for-service health delivery systems, compared to lower income countries where programmes are structured for a public health approach. Indeed, patients in high-HDI countries may be receiving equal or more comprehensive care, but at health facilities outside the HIV clinic.

Our study identified opportunities to improve the comprehensiveness of HIV care within the global IeDEA consortium. IeDEA clinics should aim to provide evidence-based services that support retention in care, including ART adherence support services, active patient outreach and peer support groups [20,34]. Almost all clinics in IeDEA reported providing one-on-one counselling for ART adherence support, but did not offer alternatives for adherence support or coordinate outreach or peer group programmes. Sites can strengthen their response to the TB-HIV epidemic by using a formal questionnaire for TB symptom screening, developing TB culture capacity, providing isoniazid preventive therapy and requiring TB-DOTS for TB management. As HIV care and treatment programmes evolve, these service gaps need to be filled through advance planning for both new and existing HIV care sites.

This work brings together and reinforces the conclusions of other studies that addressed gaps in the availability of nutritional support services [35] and TB diagnostic capacity [36] in sub-Saharan Africa, gaps in HIV prevention services [37] and paediatric HIV services globally [38], and the diverse characteristics of PEPFAR-sponsored HIV programmes [39]. The breadth of this survey, with 128 adult HIV care facilities in 41 countries worldwide, including facilities at all levels of the health care system and in both urban and rural settings, offers a rare perspective on the variability of global HIV service availability. Sites in IeDEA are part of a research network and not representative of all HIV care and treatment sites in their respective regions, or the full range of HIV clinics worldwide. Nevertheless, the diversity in survey participants allowed us to examine a wide array of programme-level characteristics.

The 96% response rate indicated that electronic surveys were effective in recruiting participating sites. Nevertheless, our study was limited as we relied on self-reported assessments of clinic services from HIV clinical providers at each site. The responses of health facility staff and survey responses were not independently verified by the research team, so we cannot rule out the possibility of facility staff over- or underreporting the availability of specific aspects of HIV services. Where possible, however, we checked the internal consistency of the survey data and resolved any discrepancies by consulting with regional data managers.

Finally, the HIV clinic survey only collected the self-reported availability of HIV prevention, care and treatment services and did not assess patient access to, routine use of and quality of these services. This limited our ability to assess actual care capacity at IeDEA sites and hence our clinic comprehensiveness metric. True comprehensive HIV care and its associated positive patient outcomes depend on accessible, high-quality clinic services. Furthermore, in developing our metric, we reviewed the HIV prevention, care and treatment services recommended by WHO and the published literature on comprehensive care. The final components of our comprehensiveness metric, however, were limited to data that had been collected in the IeDEA site survey. We intend to incorporate the most recent WHO comprehensive HIV services definition in a future iteration of the site capacity questionnaire.

## Conclusions

Data from this global site survey suggest that HIV prevention, care and treatment clinics worldwide vary greatly in capacity and that clinics more recently established in low-resource settings may offer a more comprehensive array of services onsite. Respondents reported frequently that contact tracing of patients, patient outreach, nutritional counselling, onsite viral load testing, universal TB screening and the provision of isoniazid preventive therapy were unavailable at their HIV clinics. Factors such as year of initiation of services, country HDI category and national HIV programme support from external donors such as PEPFAR appeared to contribute to the comprehensiveness of care at sites in the IeDEA network. This study provides a baseline from which to assess future changes in HIV programme structure in the context of transitions of HIV management to national authorities and decreased global funding.

## References

[1] Joint United Nations Programme on HIV/AIDS (UNAIDS) (2013). Global report: UNAIDS report on the global AIDS epidemic 2013 [Internet]. http://www.unaids.org/en/resources/campaigns/globalreport2013/globalreport/.

[2] WHO (2010). Priority interventions: HIV/AIDS prevention, treatment and care in the health sector (2010 version) [Internet]. http://whqlibdoc.who.int/publications/2010/9789241500234_eng.pdf.

[3] Fox MP, Rosen S (2010). Patient retention in antiretroviral therapy programs up to three years on treatment in sub-Saharan Africa, 2007–2009: systematic review. Trop Med Int Health.

[4] Mills EJ, Nachega JB, Buchan I, Orbinski J, Attaran A, Singh S (2006). Adherence to antiretroviral therapy in sub-Saharan Africa and North America: a meta-analysis. JAMA.

[5] Nash D, Katyal M, Brinkhof MWG, Keiser O, May M, Hughes R (2008). Long-term immunologic response to antiretroviral therapy in low-income countries: a collaborative analysis of prospective studies. AIDS Lond Engl.

[6] Braitstein P, Brinkhof MWG, Dabis F, Schechter M, Boulle A, Miotti P (2006). Mortality of HIV-1-infected patients in the first year of antiretroviral therapy: comparison between low-income and high-income countries. Lancet.

[7] Keiser O, Anastos K, Schechter M, Balestre E, Myer L, ART-LINC Collaboration of International Databases to Evaluate AIDS (IeDEA) (2008). Antiretroviral therapy in resource-limited settings 1996 to 2006: patient characteristics, treatment regimens and monitoring in sub-Saharan Africa, Asia and Latin America. Trop Med Int Health.

[8] Brinkhof MW, Dabis F, Myer L, Bangsberg DR, Boulle A, Nash D (2008). Early loss of HIV-infected patients on potent antiretroviral therapy programmes in lower-income countries. Bull World Health Organ.

[9] Brinkhof MWG, Boulle A, Weigel R, Messou E, Mathers C, Orrell C (2009). Mortality of HIV-infected patients starting antiretroviral therapy in sub-Saharan Africa: comparison with HIV-unrelated mortality. PLoS Med.

[10] Brinkhof MW, Pujades-Rodriguez M, Egger M (2009). Mortality of patients lost to follow-up in antiretroviral treatment programmes in resource-limited settings: systematic review and meta-analysis. PLoS One.

[11] Lowrance DW, Ndamage F, Kayirangwa E, Ndagije F, Lo W, Hoover DR (2009). Adult clinical and immunologic outcomes of the national antiretroviral treatment program in Rwanda during 2004–2005. J Acquir Immune Defic Syndr.

[12] Lahuerta M, Ue F, Hoffman S, Elul B, Kulkarni SG, Wu Y (2013). The problem of late ART initiation in sub-Saharan Africa: a transient aspect of scale-up or a long-term phenomenon?. J Health Care Poor Underserved.

[13] Nash D, Elul B, Rabkin M, Tun MM, Saito SM, Becker M (2009). Strategies for more effective monitoring and evaluation systems in HIV programmatic scale-up in resource-limited settings: implications for health systems strengthening. J Acquir Immune Defic Syndr.

[14] Rosen S, Fox MP (2011). Retention in HIV care between testing and treatment in sub-Saharan Africa: a systematic review. PLoS Med.

[15] Vermund SH (2011). Testing and linkage of patients to early care. AIDS.

[16] Nash D, Wu Y, Elul B, Hoos D, El Sadr W, International Center for AIDS Care and Treatment Programs (2011). Program-level and contextual-level determinants of low-median CD4+ cell count in cohorts of persons initiating ART in eight sub-Saharan African countries. AIDS.

[17] Lahuerta M, Lima J, Nuwagaba-Biribonwoha H, Okamura M, Alvim MF, Fernandes R (2012). Factors associated with late antiretroviral therapy initiation among adults in Mozambique. PLoS One.

[18] Elul B, Basinga P, Nuwagaba-Biribonwoha H, Saito S, Horowitz D, Nash D (2013). High levels of adherence and viral suppression in a nationally representative sample of HIV-infected adults on antiretroviral therapy for 6, 12 and 18 months in Rwanda. PLoS One.

[19] Adjorlolo-Johnson G, Wahl Uheling A, Ramachandran S, Strasser S, Kouakou J, Tindyebwa D (2013). Scaling up pediatric HIV care and treatment in Africa: clinical site characteristics associated with favorable service utilization. J Acquir Immune Defic Syndr.

[20] Lamb MR, El-Sadr WM, Geng E, Nash D (2012). Association of adherence support and outreach services with total attrition, loss to follow-up, and death among ART patients in sub-Saharan Africa. PLoS One.

[21] Schackman BR (2010). Implementation science for the prevention and treatment of HIV/AIDS. J Acquir Immune Defic Syndr.

[22] Padian NS, Holmes CB, McCoy SI, Lyerla R, Bouey PD, Goosby EP (2011). Implementation science for the US President's Emergency Plan for AIDS Relief (PEPFAR). J Acquir Immune Defic Syndr.

[23] Kitahata MM, Tegger MK, Wagner EH, Holmes KK (2002). Comprehensive health care for people infected with HIV in developing countries. BMJ.

[24] Van Praag E, Tarantola D, Rehle T, Saidel T, Mills S, Magnani R (2001). Evaluating care programmes for people living with HIV/AIDS. Evaluating programs for HIV/AIDS prevention and care in developing countries: a handbook for program managers and decision makers.

[25] Regional Program on AIDS/STI (2000). Building blocks: comprehensive care guidelines for persons living with HIV/AIDS in the Americas: summary report [Internet]. http://www1.paho.org/English/AD/FCH/AI/BuildingBlocks.pdf.

[26] McGowan CC, Cahn P, Gotuzzo E, Padgett D, Pape JW, Wolff M (2007). Cohort profile: Caribbean, Central and South America Network for HIV research (CCASAnet) collaboration within the International Epidemiologic Databases to Evaluate AIDS (IeDEA) programme. Int J Epidemiol.

[27] Egger M, Ekouevi DK, Williams C, Lyamuya RE, Mukumbi H, Braitstein P (2012). Cohort profile: the international epidemiological databases to evaluate AIDS (IeDEA) in sub-Saharan Africa. Int J Epidemiol.

[28] Gange SJ, Kitahata MM, Saag MS, Bangsberg DR, Bosch RJ, Brooks JT (2007). Cohort profile: the North American AIDS cohort collaboration on research and design (NA-ACCORD). Int J Epidemiol.

[29] Dabis F, Balestre E, Braitstein P, Miotti P, Brinkhof WGM, Schneider M (2005). Cohort profile: antiretroviral therapy in lower income countries (ART-LINC): international collaboration of treatment cohorts. Int J Epidemiol.

[30] Divaris K, Newman J, Hemingway-Foday J, Akam W, Balimba A, Dusengamungu C (2012). Adult HIV care resources, management practices and patient characteristics in the phase 1 IeDEA Central Africa cohort. J Int AIDS Soc.

[31] Harris PA, Taylor R, Thielke R, Payne J, Gonzalez N, Conde JG (2009). Research electronic data capture (REDCap) – a metadata-driven methodology and workflow process for providing translational research informatics support. J Biomed Inform.

[32] International human development indicators – UNDP [Internet] http://hdrstats.undp.org/en/indicators/103106.html.

[33] Department of State (2011). The Office of Electronic Information B of PA. FY 2008 PEPFAR Country Operational Plans [Internet]. http://www.pepfar.gov/countries/cop/2008/index.htm.

[34] Geng EH, Nash D, Kambugu A, Zhang Y, Braitstein P, Christopoulos KA (2010). Retention in care among HIV-infected patients in resource-limited settings: emerging insights and new directions. Curr HIV/AIDS Rep.

[35] Anema A, Zhang W, Wu Y, Elul B, Weiser SD, Hogg RS (2012). Availability of nutritional support services in HIV care and treatment sites in sub-Saharan African countries. Public Health Nutr.

[36] Saito S, Howard AA, Reid MJA, Elul B, Scardigli A, Verkuijl S (2012). TB diagnostic capacity in sub-Saharan African HIV care settings. J Acquir Immune Defic Syndr.

[37] Spaar A, Graber C, Dabis F, Coutsoudis A, Bachmann L, McIntyre J (2010). Prioritising prevention strategies for patients in antiretroviral treatment programmes in resource-limited settings. AIDS Care.

[38] IeDEA Pediatric Working Group (2013). A survey of paediatric HIV programmatic and clinical management practices in Asia and sub-Saharan Africa – the International Epidemiologic Databases to Evaluate AIDS (IeDEA). J Int AIDS Soc.

[39] Filler S, Berruti AA, Menzies N, Berzon R, Ellerbrock TV, Ferris R (2011). Characteristics of HIV care and treatment in PEPFAR-supported sites. J Acquir Immune Defic Syndr.

